# Influence of Mechanical Skin Treatments on Dermal Penetration Efficacy of Active Ingredients

**DOI:** 10.3390/pharmaceutics14091788

**Published:** 2022-08-26

**Authors:** Cornelia M. Keck, Em-on Chaiprateep, Henriette Dietrich, Soma Sengupta

**Affiliations:** Department of Pharmaceutics and Biopharmaceutics, Philipps Universität Marburg, 35037 Marburg, Germany

**Keywords:** massage, corneotherapy, cosmetic, facial treatment, professional skin care, skin, dermal drug delivery, penetration enhancement

## Abstract

The effective dermal penetration of active ingredients (AI) is a major task in the formulation of topical products. Besides the vehicle, the mechanical skin treatments are also considered to impact the penetration efficacy of AI. In particular, professional skin treatments, i.e., professional cosmetic skin treatments, are considered to be optimal for the dermal delivery of AI. However, a systematic study that proves these theories is not yet available and was therefore performed in this study while utilizing an ex vivo porcine ear model with subsequent digital image analysis. Hydrophilic and lipophilic fluorescent dyes were used as AI surrogates and were applied onto the skin without and with professional skin treatments. The skin hydration and the penetration efficacy were determined, respectively. Results showed that professional skin treatments with massage were able to increase the skin hydration, whereas a professional skin treatment without massage could not increase the skin hydration when compared to skin without professional skin treatment. Regarding the penetration efficacy, it was found that all parameters tested, i.e., type of professional skin treatment, lipophilicity of the AI, and the time point at which the AI are applied onto the skin, can have a tremendous impact on the penetration efficacy of the AI. The most effective penetration and the most effective skin hydration is achieved with a professional skin treatment that includes a professional skin massage. This kind of skin treatment can therefore be used to improve dermal drug delivery.

## 1. Introduction

**Figure 1:**
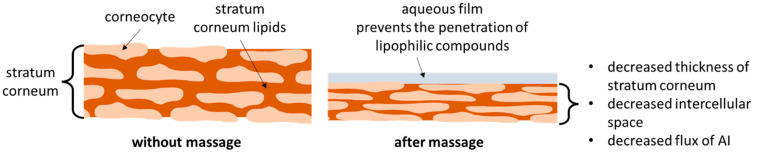
Influence of skin massage on stratum corneum properties.

Dermal drug delivery is a major administration route, and in most cases, liquid or semi-solid formulations are used for this. Today, it is well known that the vehicle has a major influence on the penetration efficacy of active ingredients (AI) [1,2,3,4,5]. However, only a few studies are available on the influence of mechanical skin treatments in the dermal penetration efficacy of AI [6,7]. The results of these studies confirmed that mechanical treatments, e.g., tape stripping, microdermabrasion or microneedeling, that cause a rupture or (partial) removal of the stratum corneum (SC), can increase the penetration of active ingredients into the skin [6]. Interestingly, the results also showed that massage can decrease the dermal penetration efficacy of AI [6,7]. The reason for the decreased penetration efficacy is the pressure during the massage that causes an increase in the density of the SC. The higher density is considered to reduce the diffusion coefficient of the AI and thus the flux of the AI into and through the SC (Figure 1). Moreover, it was found that the pressure during massage can squeeze out water from the skin. The squeezed-out water can form a hydrophilic film on top of the skin, which represents a barrier for lipophilic fluids and AI, and thus prevents the penetration of hydrophobic AI (Figure 1). On the other hand, it can promote the penetration of hydrophilic AI [7].

Despite the results of the above-mentioned studies, today, massage is often considered to improve dermal drug delivery [8,9,10]. Hence, often, skin massage is recommended for optimal uptake of the AI into the skin. This is not only true for pharmaceutical applications, but also for professional cosmetic skin care in beauty salons. In general, a professional cosmetic treatment is considered to allow for more effective penetration of AI into the skin than the application of AI without any mechanical skin treatment [11]. During a professional cosmetic treatment, which is composed of different treatment steps, massage is considered to be the essential treatment step for the effective incorporation of the AI into the skin [12]. Interestingly, a study that provides scientific evidence for the efficacy of massage during a professional cosmetic skin treatment is not yet available. A study that investigates the influence of a professional cosmetic skin treatment on the dermal penetration of AI in comparison to a classical application (no professional skin treatment) is also not yet available. Thus, today it is not known which type of mechanical skin treatment should be used for optimal dermal delivery of AI. The aim of this study was therefore to systematically investigate if a professional skin treatment enables an improved penetration of AI into the skin. Secondly, it aimed at investigating the effect of the massage during the professional skin treatment on the dermal penetration efficacy of AI.

**Figure 2:**
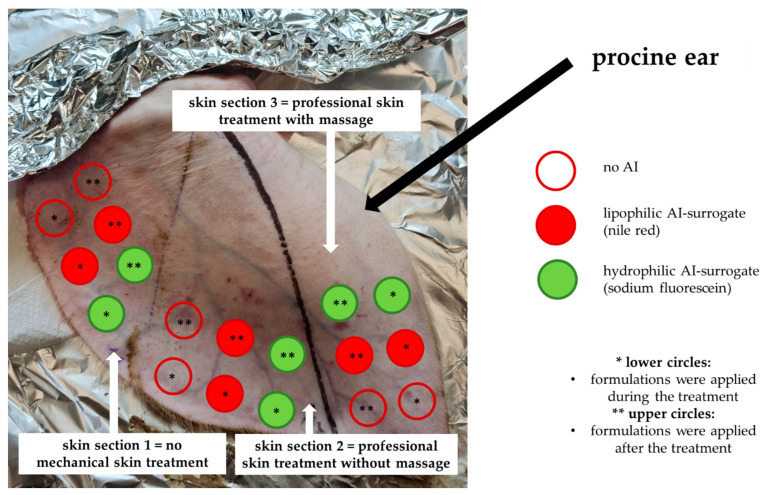
Scheme of study design.

The study was performed on an ex vivo porcine ear model [13]. A hydrophilic dye (sodium fluorescein) was used as hydrophilic AI surrogate, and a lipophilic fluorescent dye (nile red) was used as lipophilic AI surrogate. The porcine ears were divided into three sections (Figure 2). Skin Section 1 was used as control and underwent no professional skin treatments. Hence, the AI surrogates were applied on the skin without professional skin treatment and without massage. Skin Section 2 underwent a professional skin treatment without massage, and Skin Section 3 underwent a professional skin treatment with massage (Figure 2). A professional skin treatment involves different steps, i.e., welcome mask, cleansing, peeling, application of highly active AI with a mask and a subsequent professional skin massage. At the end of the professional treatment, the skin is treated with professional skin care products that help (i) to deliver AI deep into the skin and (ii) to maintain the skin barrier function [12]. This means that during a professional skin treatment, AI are applied at two different time points. The first application of AI are during the treatment (mask), and the second application of AI takes place after the treatment (AI in professional skin care products). Therefore, also in this study, the AI were applied at two different time points, i.e., during the treatment and after the treatment (Figure 2, Table 1).

## 2. Materials and Methods

### 2.1. Materials

The AI surrogates sodium fluorescein (SF) and nile red (NR) were purchased from Carl Roth GmbH (Karlsruhe, Germany) and Sigma-Aldrich Chemie GmbH (Steinheim, Germany), respectively. Medium chain triglycerides (Miglyol^®^ 812) were purchased from Caesar & Loretz GmbH (Hilden, Germany), and purified water was freshly obtained from a PURELAB^®^Flex 2 water purification system (ELGA Labwater, Veolia Water Technologies Deutschland GmbH, Celle, Germany).

### 2.2. Methods

#### 2.2.1. Preparation of the Formulations

The hydrophilic AI surrogate was dissolved in water to contain 0.005% (*w*/*w*) SF and the lipophilic AI surrogate was dissolved in Miglyol to contain 0.005% (*w*/*w*) NR.

#### 2.2.2. Preparation of the Porcine Ears and Performance of Professional Skin Care Treatments

**Figure 3:**
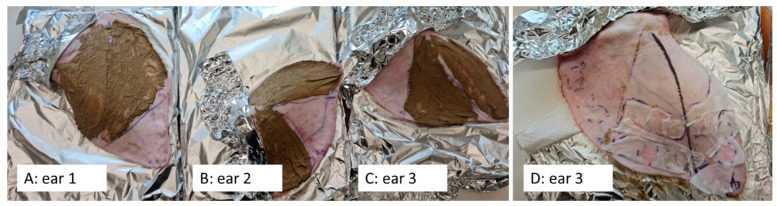
Porcine ears during professional cosmetic skin treatment, i.e., (**A**–**C**): ears during the peeling procedure, (**D**): ear after application of AI during treatment and after covering the skin with a towel mask.

The porcine ears were freshly obtained from a local slaughterhouse, washed with lukewarm water and were carefully dabbed dry with lint-free tissue. The ears were separated into three areas (cf. Figure 2). Afterward, the professional cosmetic treatment was performed in the Skin Section 2 and Section 3. The professional skin treatment was started by applying a tissue that was wetted with hot water on top of the porcine skin (welcome mask [12]). The tissue was removed after about 30 s, and the skin was cleansed with cleanser towels (ISANA Reinigungstücher 5 in 1, Dirk Rossmann GmbH, Burgwald Germany). After the cleansing, the skin underwent a peeling. The peeling formulation was composed of a 1:1 mixture of peeling particles (loess powder, Luvos healing earth, Heilerde-Gesellschaft Luvos Just GmbH & Co. KG, Friedrichsdorf, Germany) and a peeling gel (ISANA Peeling Gel, Dirk Rossmann GmbH, Burgwald Germany). The peeling formulation was applied on the skin and left until dry for about 30 min (Figure 3A–C). Afterward, the peeling formulation was removed with towels that were wetted with hot water. The skin was dabbed dry, and the AI formulations were applied on the skin (10 µg/cm^2^) with a saturated glove without massage.

Immediately after application of the AI formulations, the skin was covered with a towel mask (ISANA Tuchmaske Collagenbooster, Dirk Rossmann GmbH, Burgwald Germany, Figure 3D). The mask was left on the skin for 30 min before it was removed. The next step was the professional skin massage that was applied only for Skin Section 3 (cf. Figure 2). The massage was done with an amphiphilic cream formulation (Schaebens Anti-Falten Maske, Haus Schaeben GmbH & Co. KG, Frechen, Germany) that was gently massaged into the skin with a gloved finger by applying soft pressure in circles and in rotating directions for 3 min. Finally, the AI formulations after the treatment were applied on the skin. After 60 min of incubation time at room temperature, the experiment was terminated, and skin punch biopsies (Ø 10 mm) were taken from each skin area (cf. Figure 2, Table 1). The punches were embedded in Tissue-Tek^®^ (Sakura Finetek Europe B.V., Alphenaan den Rijn, The Netherlands) and frozen until further use. The experiment was performed in triplicate on three independent porcine ears, i.e., on ears from different pigs.

#### 2.2.3. Determination of Skin Hydration and Transepidermal Water Loss (TEWL)

Professional cosmetic skin treatments are considered to improve the skin hydration and to maintain and restore the skin barrier function [14]. To date, the influence of a professional skin treatment on skin hydration and TEWL, which is a measure for the skin barrier integrity, was not yet assessed on porcine ears. Therefore, to allow for a link between human in vivo data and the ex vivo porcine ear model, the TEWL and skin hydration were also assessed in this study. TEWL and skin hydration were assessed prior to the beginning of the professional treatment, after the peeling, and at the end of the experiment, i.e., after 1 h penetration time of the AI that was applied at the end of the professional skin treatments (c.f. Section 2.2.2, Table 1). The TEWL was assessed with a Tewameter^®^ (TM 300, Courage + Khazaka electronic GmbH, Köln, Germany), and the skin hydration was determined with a Corneometer^®^ (CM 825, Courage + Khazaka electronic GmbH, Köln, Germany). All measurements were performed in triplicate on each skin section.

#### 2.2.4. Determination of Penetration Efficacy

The dermal penetration efficacy was assessed from 20 µm thick, vertical skin cuts that were obtained from the skin punch biopsies with a cryomicrotome (Mod. 2700 Reichert-Jung, Nußloch, Germany). The skin cuts were imaged with inverted epifluorescence microscopy (Olympus CKX53, equipped with an Olympus DP22 color camera, Olympus LifeScience Solutions GmbH, Hamburg, Germany) with 200-fold magnification. The light source (130 W U-HGLGPS illumination system, Olympus Deutschland GmbH, Hamburg, Germany) was set to 50%, and the exposure time was 50 ms. The filter was the DAPI HC filter block system (excitation filter: 460–490 nm (BP), dichroic mirror 500 nm, emission filter: starting at 500 nm (LP) for the SF and excitation filter: 540–560 nm (BP), dichroic mirror 570 nm, emission filter: starting at 580 nm (LP) for the NR). From each biopsy, at least 12 skin cuts and 40 images were obtained, which resulted in a total of at least 120 images for each formulation tested (n = 3).

**Figure 4:**
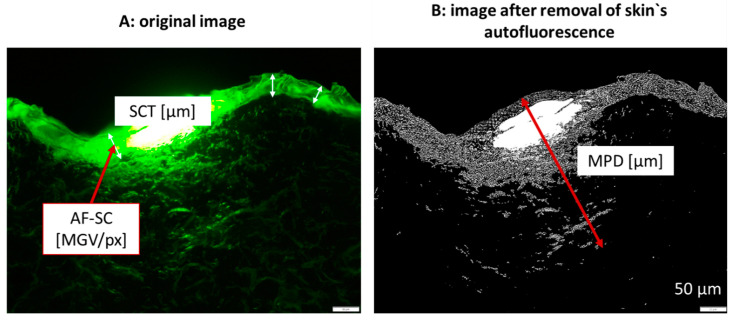
Scheme for assessing the dermal penetration efficacy of AI with the ex-vivo porcine ear model with subsequent image analysis. (**A**) The stratum corneum thickness (SCT) and the autofluorescence of the stratum corneum (AF-SC) are determined form the original microscopic images obtained from inverted epifluorescence microscopy. (**B**) The mean penetration depth (MPD) and the intensity (MGV/px) after the removal of the skin’s autofluorescence (AROSA value) are determined from the images after automated thresholding that subtracts the autofluorescence of the skin from the image (cf. Supplementary Materials Table S1). The remaining pixel surrogate the amount of penetrated AI [13].

The images obtained were subjected to digital image analysis as described earlier by using Image J software version 153 k [4,7,15,16,17]. The stratum corneum thickness (SCT) was determined directly from the original images with the scale function of the software, while setting the scale to 2.84 µm/px. The SCT measurements yielded the thickness of the SC and allowed also to determine the autofluorescence of the SC (AF-SC), which is represented by the measured light intensity/px within the measured SCT line (Figure 4A).

Similar to the SCT, the mean penetration depth (MPD) of the AI surrogates was determined. This was conducted using the images that were subjected to an automated threshold algorithm which subtracted the autofluorescence of the skin from the image (Figure 4B and Supplementary Materials Table S1). After the removal of the skin’s autofluorescence (AROSA value), the remaining mean grey value/pixel [MGV/px] within the image can be considered to surrogate the total amount of penetrated AI [13]. Therefore, the AROSA value and the MPD were used as effective parameters to compare the penetration efficacy of the AI surrogates from the differently treated skin sections. For a better comparison, the values obtained were also transferred into relative values, where the untreated skin, i.e., the values obtained from the skin without professional skin treatment, were set to 100%. A list of abbreviations is provided in the Supplementary Material section (cf. Supplementary Materials Table S2).

#### 2.2.5. Statistical Analysis

Descriptive statistics (mean values, standard deviations) and the comparison of the mean values were assessed with JASP software version 16.2 (Universiteit van Amsterdam, Amsterdam, The Netherlands) [18]. Normal distribution was tested with the Shapiro–Wilk test. Variance homogeneity was tested with the Levene’s test. ANOVA (normally distributed data with Welch adaption in case of variance inhomogeneity) and Kruskal–Wallis tests (non-parametric data) were performed with appropriate post hoc tests (Tukey, Games–Howell or Dunn’s post hoc test [19]). Where appropriate, t tests were also performed to determine significant differences between two distinct mean values. All *p*-values < 0.05 were considered statistically significant. Results are shown as mean values ± standard deviation (SD). Relative values are shown as mean ± relative standard deviation (RSD). In the Figures, significant differences between the different means are indicated by * *p* < 0.05, ** *p* < 0.01, *** *p* < 0.001, respectively.

## 3. Results and Discussion

### 3.1. Influence of Professional Skin Treatment on Bio-Physical Skin Properties

**Figure 5:**
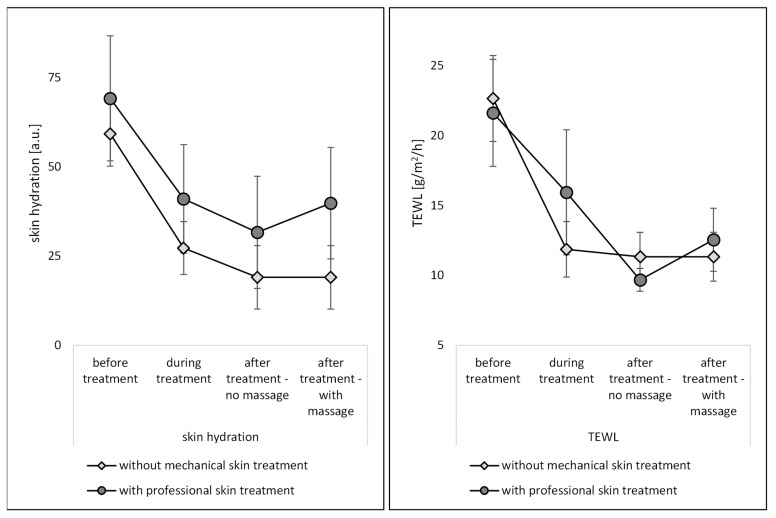
Influence of mechanical skin treatments on skin hydration (**left**) and transepidermal water loss (TEWL) (**right**).

The ex vivo measurements were performed with skin probes (Corneometer and Tewameter) that are typically applied in vivo, i.e., in humans. Hence, the data obtained in this study can also be linked to in vivo studies. The skin probes were used to assess the TEWL and the skin hydration. The skin hydration measurements showed that the skin of the pig ears dries out during the experiment (Figure 5). The skin hydration of the skin without professional skin treatment was reduced to about 50% after the first part of the skin treatment (after peeling) and was reduced to approximately 33% at the end of the experiment. The TEWL, indicating how much water is evaporating from the skin per time [20], was reduced to 50%. In contrast to non-treated skin, professional skin treatments could significantly increase the skin hydration (Figure 4—left). The effect was most pronounced during the treatment and after the treatment when massage was applied. The professional skin treatment without massage was found to result in a less efficient skin hydration. The effect was not expected, because a previous study showed that massage squeezes out water from the skin, which results in a reduced skin hydration. The oppose results in this study, i.e., massage increased the skin hydration, might be explained by the fact that the entire professional skin treatment in this study acts differently on the skin than an isolated massage. Isolated massage was found to squeeze the skin, which results in an increased density of the SC and a water layer on top of the SC [6,7]. In this study, the skin was professionally cleansed and peeled, which hydrated the skin. The next step was the massage of the skin with a cream, which was performed for some of the skin areas, whereas others were left without massage and without the application of the cream. In the case of the hydrated skin that was massaged, the massage might have resulted in an increase in skin density within the upper layers of the SC (cf. Figure 1). In addition, it can be assumed that the cream or parts of the cream were incorporated into the skin during the massage. In this way, the water from the cream can be considered to cause a direct hydration of the skin, whereas the oil from the cream might have caused an indirect hydration due to its occlusive properties. Hence, oil from the cream that penetrated into the skin can be considered to “block” the channels in the SC through which water from the skin can evaporate. Due to the “smaller channels” within the SC, the water from deeper layers in the skin could not evaporate anymore, whereas for the skin that was not massaged, the “water channels” within the SC were left open, which allowed the water to evaporate over time. This resulted in a lower skin hydration at the end of the experiment and caused also lower TEWL values, i.e., less remaining water for evaporation within the skin (Figure 5—right).

**Figure 6:**
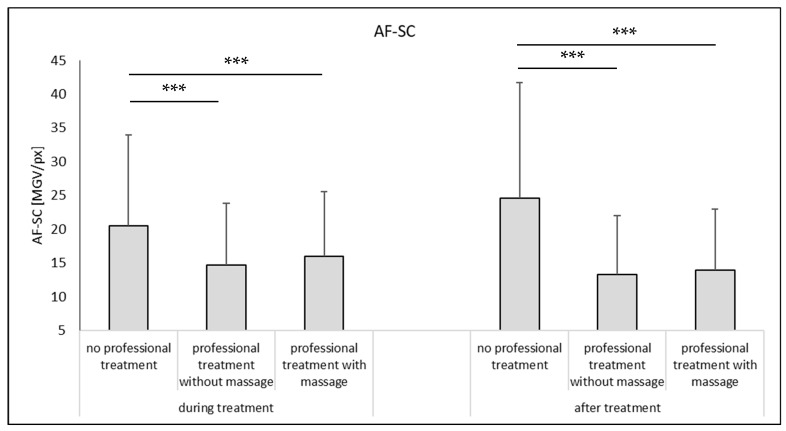
Influence of mechanical skin treatments on the autofluorescence of stratum corneum (AF-SC) from skin without AI treatment. *** *p*-value < 0.001.

The data obtained from the skin probes were completed with the skin parameters obtained from the microscopic images from epifluorescence microscopy after digital image analysis (Figure 6). During the experiment, the autofluorescence of the stratum corneum (AF-SC) increased for the non-treated skin (approx. +20%, Mann–Whitney test, *p* < 0.05). An increase in AF-SC is caused by an increase in scattered light that is often caused by a decrease in the hydration of the SC [21]. Hence, the data obtained from image analysis substantiate the data obtained from the bio-physical skin parameters. The AF-SC decreased during the professional skin treatment. The reduction is caused by the removal of sebum and bacteria, which possess a strong autofluorescence [22]. In addition, a decrease in AF-SC might also be caused by an increase in skin hydration [21,23]. Hence, water within the SC reduces its optical density and therefore the measured AF-SC. The professional skin treatment with massage resulted in a slightly higher AF-SC, indicating—as mentioned above—an increase in optical density, probably due the squeezing of the skin.

**Figure 7:**
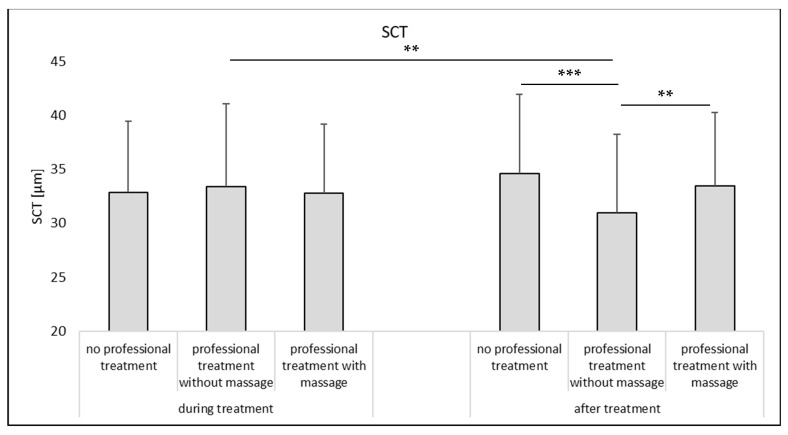
Influence of mechanical skin treatments on the stratum corneum thickness (SCT) from skin without AI treatment. ** *p*-value < 0.01, *** *p*-value < 0.001.

The SCT is considered as an alternative surrogate for skin hydration, i.e., high SCT-values indicate high skin hydration and vice versa [15,16]. During the treatment, no differences in SCT were determined between non-treated and treated skin (Figure 7—left) and also after the treatment no significant differences in SCT were found between the non-treated skin areas and the professionally treated skin with massage (Figure 7—right). However, a slight but significant trend toward a lower SCT was found for the skin that underwent a professional skin treatment without massage. Data, therefore, also indicate that a professional skin treatment without massage results in a less hydrated SC at the end of the treatment.

Based on the data obtained in this part of the study, it can be concluded that mechanical skin treatments that are applied on the skin during a professional skin treatment increase the skin hydration. A professional skin treatment without massage is less beneficial when compared to a professional skin treatment with massage but results still in a significantly higher skin hydration when compared to skin without professional skin treatment (cf. Figure 5, Welch test, *p* < 0.001).

### 3.2. Influence of Mechanical Skin Treatments on Dermal Penetration Efficacy

**Figure 8:**
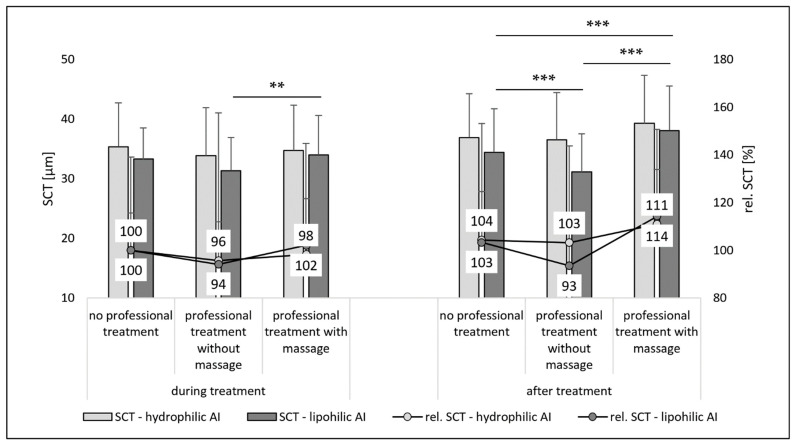
Influence of mechanical skin treatments on the stratum corneum thickness (SCT) from skin biopsies treated with AI formulations. ** *p*-value < 0.01, *** *p*-value < 0.001.

**Figure 9:**
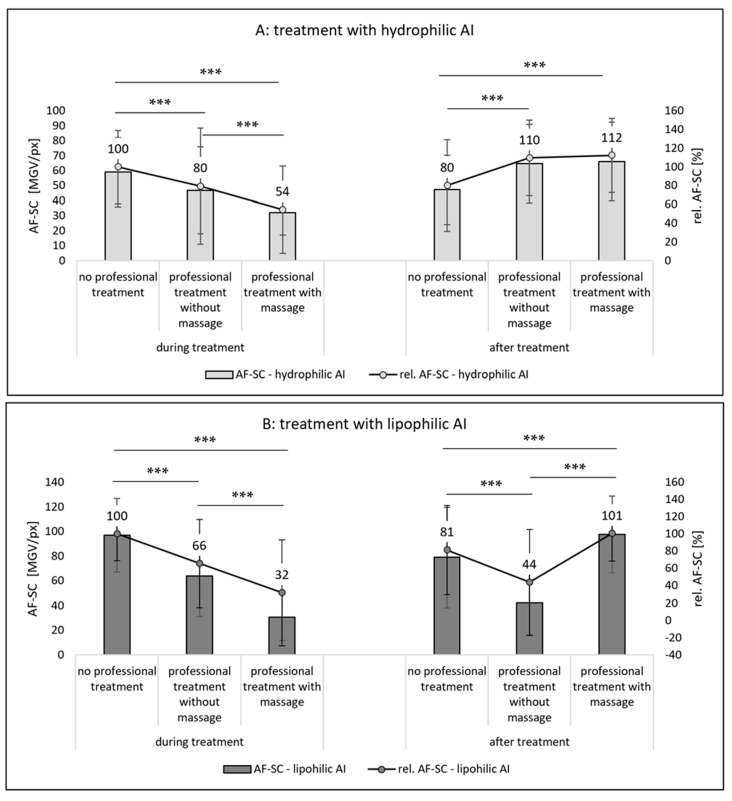
Influence of mechanical skin treatments on the autofluorescence of stratum corneum (AF-SC) from skin biopsies treated with AI formulations. (**A**) Skin treated with hydrophilic AI-surrogate, (**B**) skin treated with lipophilic AI surrogate. *** *p*-value < 0.001.

The influence of the dermal penetration efficacy was determined for a hydrophilic AI surrogate and for a lipophilic AI surrogate, respectively. The penetration parameters assessed were the AROSA value that surrogates the total amount of penetrated AI and the MPD which indicates the penetration depth of the AI into the skin. In addition, also the SCT and the AF-SC were determined from the skin sections treated with the hydrophilic and lipophilic AI surrogates (Figure 8 and Figure 9).

Upon application of the AI solutions, i.e., water with SF and oil with NR, the changes in SCT due to professional treatments were more pronounced than for the skin where no AI formulations were applied (cf. Figure 7 and Figure 8). The SCT reducing effect of a professional skin treatment without massage was significant for the formulations with the lipophilic AI, and, also for all AI-treated samples, the skin hydrating effect of the professional skin treatment with massage was significant (Figure 8). With this, the data are in line with the results obtained from the skin probe measurements (cf. Figure 5). Data therefore provide further evidence and substantiate that a professional cosmetic skin treatment with massage increases skin hydration.

The autofluorescence of the stratum corneum was also assessed from the skin sections that were treated with AI. In contrast to the skin without AI treatment, the results of the AI-treated sections represent not only the AF-SC, but a sum of the AF-SC and the amount of AI that penetrated into the SC (Figure 9). If the AI are applied during the professional skin treatment, the penetrated amount of AI in the SC is significantly less when compared to the application of AI on skin without professional skin treatment (Figure 9A,B—left). The effect is less pronounced for professional skin treatments without massage and more pronounced for the professional skin treatment with massage. Additionally, the effect was more pronounced for the lipophilic AI surrogate than for the hydrophilic AI surrogate (Figure 9A,B—left).

When the AI was applied after the professional skin treatment, the professional skin treatment with massage was found to be beneficial for the dermal penetration efficacy of the AI (Figure 9A,B—right). However, a professional skin treatment without massage was only beneficial for the hydrophilic AI (Figure 9A—right) but had a penetration reducing effect for the hydrophobic AI (Figure 9B—right). Hence, data indicate that both, the professional treatment and the time point at which the AI are applied on the skin, i.e., during or after the professional skin treatment, influence the dermal penetration efficacy of AI. From the data obtained, it becomes obvious that a professional skin treatment with included massage modulates and hydrates the SC, which then enables an improved dermal penetration efficacy of AI into the SC. However, the effect can only be observed if the AI are applied onto the skin after the skin treatment is finished. Application of AI during the professional skin treatment is less effective when compared to skin without professional skin treatment. The reason for this is considered to be mainly related to the longer penetration time of the AI for the skin without professional skin treatment (cf. Section 2.2.2). For skin without professional skin treatment, the AI were applied on the skin and remained there until the end of the experiment. In case of the skin that underwent the professional skin treatments, the formulations were applied underneath a facial mask that was removed after 30 min. The reduced amount of penetrated AI that were applied during this treatment therefore indicates that parts of the AI might have also penetrated into the mask. The even further reduced AI penetration for the skin that was massaged (Figure 9A,B—left) indicates that the cream that was used with the massage and the pressure during the massage might have rubbed away some of the remaining AI from the skin. In fact, the application of AI during the professional skin treatment was found to be not effective when compared to the application of AI on skin without professional skin treatment. In this way, professional skin treatment without massage reduced the penetration efficacy of the hydrophilic AI surrogate into the SC to about 80% and the penetration efficacy into the SC of the lipophilic AI surrogate to about 66%. The professional skin treatment with massage reduced the penetrated amount of AI to 54% for the hydrophilic AI and to only 32% in case of the lipophilic AI (Figure 9A,B—left).

**Figure 10:**
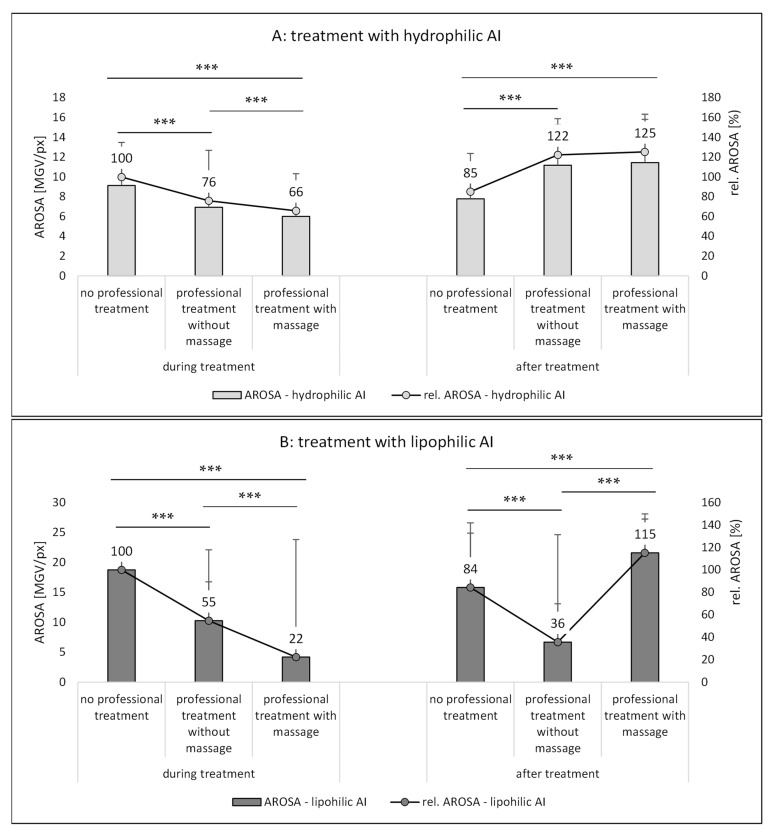
Influence of mechanical skin treatments on the total amount of penetrated AI (measured as intensity (MGV/px) **a**fter the **r**emoval **o**f the **s**kin’s autofluorescence—**A**ROSA). (**A**) Skin treated with hydrophilic AI surrogate, (**B**) skin treated with lipophilic AI surrogate. *** *p*-value < 0.001.

**Figure 11:**
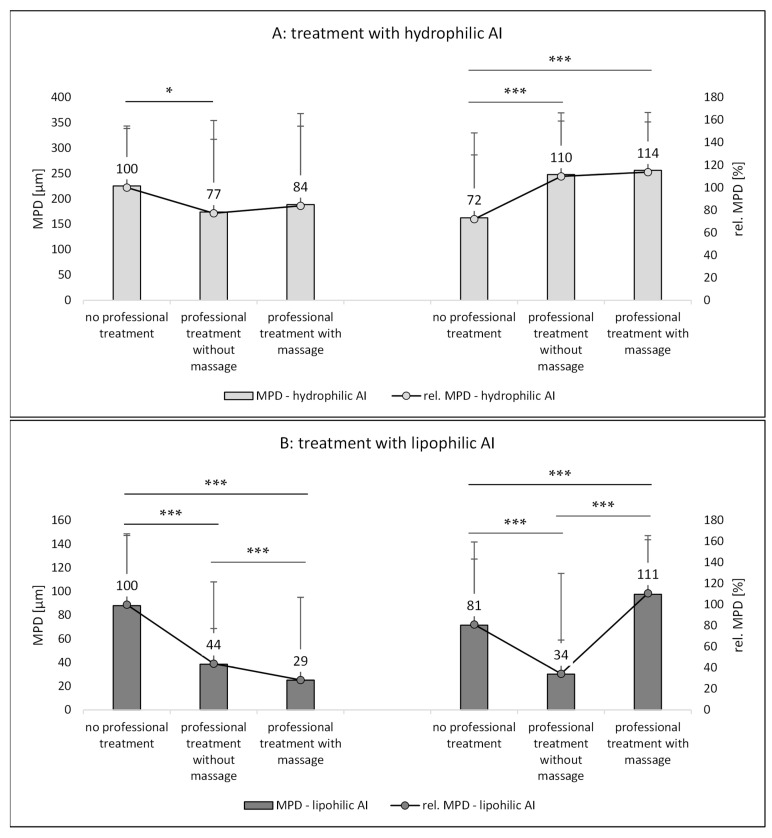
Influence of mechanical skin treatments on the mean penetration depth (MDP) of AI. (**A**) Skin treated with hydrophilic AI surrogate, (**B**) skin treated with lipophilic AI surrogate. * *p*-value < 0.05, *** *p*-value < 0.001.

The trends found from the AF-SC measurements were also seen for the other two penetration parameters, i.e., the AROSA values and the MPD (Figure 10 and Figure 11). Based on the data, it can therefore be concluded that application of AI during a professional skin treatment is not optimal because the penetration of AI is less effective when compared to the application without professional skin treatment. In addition, it is concluded that a professional cosmetic treatment should be performed with massage. This will allow for the most effective penetration of both hydrophilic and lipophilic AI if they are applied after the professional skin treatment (Figure 9A,B—right, Figure 10A,B—right, and Figure 11A,B—right). Skin massage seemed to be especially important for the effective penetration of lipophilic AI (Figure 9B—right, Figure 10B—right, and Figure 11B—right). A possible explanation for this observation might be the fact the massage was performed together with a cream. The skin hydration measurements already indicated that the massage with the cream increased the skin hydration when compared to skin that underwent a professional skin treatment without massage, and it was speculated that massage might have incorporated some hydrophobic compounds that close “pores” of the skin and thus prevent water loss from inner parts of the skin (cf. Section 3.1).

Based on these considerations, it can also be speculated that the massage with the cream created a more hydrophobic SC (or skin surface/environment) than the treatment without cream and massage. When considering the results of the two abovementioned studies [6,7] that showed that massage can create an aqueous film on top of the skin that prevents the penetration of lipophilic AI (cf. Figure 1), it can be assumed that also other mechanical treatments, i.e., washing, cleansing and peeling, hydrate the skin, which then results in the formation of a “water front” that hampers the penetration of lipophilic AI. In this study, the lipophilic AI was dissolved in oil. As oil does not mix with water, it is highly likely that the oil was added on top of this waterfront. The lipophilic AI surrogate—which is easily soluble in the oil but sparingly soluble in water—then preferred to remain in its preferred solvent (oil), which resulted in the observed poor penetration of the AI. In contrast, massage with the oil-containing cream can be considered to remove the waterfront on top of the skin and might even be able to incorporate some lipophilic compounds from the cream into the SC [7]. This results in a more lipophilic skin surface into which the lipophilic AI could penetrate more easily (Figure 9B—right, Figure 10B—right, and Figure 11B—right).

The data obtained for the hydrophilic AI support this theory. Here, the influence of massage is almost negligible (Figure 10A—right, and Figure 11A—right). The reason is that the waterfront on top of the skin will not hamper but rather accelerate the penetration of the hydrophilic AI into the skin. However, also for the hydrophilic AI, the penetration is slightly less for the professionally treated non-massaged skin when compared to the professionally treated massaged skin. The reason is the dehydration of the skin during the experiment, which reduces the penetration of the hydrophilic AI over time. Massage was found to improve the water retention of the skin (cf. Section 3.1.). Hence, over time, good skin hydration is longer maintained for the massaged skin and thus the total penetration of the hydrophilic AI is higher when the skin is treated professionally with skin massage.

Besides the total amount of penetrated AI, it is also interesting to investigate if the AI are penetrating into the upper layers of the SC, or if they are reaching deeper layers of the viable skin. This information can be assessed by comparing the penetration depth (MPD) of the AI to the SCT. If the MPD is >SCT, the AI was able to penetrate through the SC and if the MPD is larger than the epidermis the formulation can be considered to allow for a transdermal penetration of the AI. The MPD of the hydrophilic AI, independent of the type of skin treatment, was always >150 µm (Figure 11A). The mean thickness of the epidermis of porcine ears is in the range of about 100–110 µm and thus comparable to the thickness of the human epidermis [24]. Hence, in this study, in all cases, a transdermal penetration of the hydrophilic AI was achieved.

In contrast, for the lipophilic AI, the MPD was about 80 µm without professional skin treatment (Figure 11B). Hence, without professional skin treatment, the AI penetrated through the SC into the viable layers of the epidermis. Professional skin treatments with massage and application of the AI afterwards could increase the penetration depth (Mann–Whitney test, *p* < 0.05). The application of the lipophilic AI onto skin after a professional treatment without massage and the application during the professional skin treatment reduced the MPD to below the SCT. Hence, in these cases, the penetration of the lipophilic AI was not deeper than the SC. Therefore, data of the study also show that mechanical treatments modulate not only the total amount of penetrated AI, but also the skin side that is reached by the AI. This fact is also important to note because the activity and efficacy of an AI depends on both the total amount of penetrated AI and the penetration depth because only AI that reach the desired target side in a sufficient dose and time can unfold their pharmacodynamic potential. Therefore, the type of skin treatment and the application time of the AI, either during or after the professional skin treatment, must be considered to be important parameters for the efficacy of AI-loaded dermal products.

## 4. Conclusions

Mechanical skin treatments can influence the skin hydration and the penetration efficacy of AI. A professional, cosmetic skin treatment involves washing, cleansing and peeling of the skin. Afterwards AI are applied on the skin and the skin is covered with a mask. After an adequate penetration time, the mask is removed, and the skin is massaged subsequently. Finally, the skin is treated with professional products for optimized delivery of AI. This study confirmed that this kind of professional skin treatment increases the skin hydration. In this sense, massage was found to be essential for a long-lasting skin hydration. The professional skin treatment was also shown to influence the dermal penetration efficacy of active ingredients. For effective dermal penetration, the AI should be applied to the skin at the end of the professional skin treatment. Massage was found to be not mandatory for the effective penetration of hydrophilic AI but was identified to be an important mechanical treatment step if an effective dermal penetration of lipophilic AI is desired. If these parameters are considered, a professional skin treatment can significantly enhance the dermal penetration efficacy of AI. However, if the parameters are not considered, a professional skin treatment can also cause a less efficient dermal penetration of AI. Results of the study provide a base for the development of optimized skin treatments that allow for efficient skin hydration, skin care and highly efficient penetration of AI at the same time. Based on the outcome of this study, a professional cosmetic treatment with massage can be considered to be optimal for effective dermal drug delivery and can therefore be recommended to be used for improved dermal delivery of AI—not only for the improved delivery of cosmetic AI, but also for the improved efficacy of topical pharmaceutical formulations.

## Figures and Tables

**Table 1:** Overview of mechanical skin treatments that were performed during the professional skin treatment on the different skin sections of the porcine ears (cf. Figure 2).

No. of Treatment Step	Type of Treatment	Skin Section		
		1	2	3
1	welcome mask (approx. 30 s)	-	x	x
2	cleansing procedure (approx. 3 min)	-	x	x
3	peeling (approx. 30 min)	-	x	x
4	application of AI formulations during the treatment	x	x	x
5	application of towel mask (30 min)	-	x	x
6	skin massage (3 min)	-	-	x
7	application of AI formulations after the treatment	x	x	x

## Data Availability

Not applicable.

## Supplementary Materials

The following supporting information can be downloaded at: https://www.mdpi.com/article/10.3390/pharmaceutics14091788/s1, Table S1: macros used for automated threshold algorithms. Table S2: List of Abbreviations.
