# Supplementary material for: Influence of Mechanical Skin Treatments on Dermal Penetration Efficacy of Active Ingredients
Source: Pharmaceutics. 2022 Aug 26;14(9):1788. doi: 10.3390/pharmaceutics14091788 (PMC9502347; doi:10.3390/pharmaceutics14091788)
Supplement: Supplementary file 1 [file pharmaceutics-14-01788-s001.zip › pharmaceutics-1863114-supplementary.pdf]

pharmaceutics

MDPI

Article

Influence of mechanical skin treatments on dermal penetration efficacy of active ingredients

Cornelia M. Keck *, Em-on Chaiprateep, Henriette Dietrich, and Soma Sengupta

Department of Pharmaceutics and Biopharmaceutics, Philipps Universität Marburg, 35037 Marburg, Germany
* Correspondence: cornelia.keck@pharmazie.uni-marburg.de; Tel.: +49 6421 282 5881

Supplemantary material:

Table S1: macros used for automated threshold algorithms. Left: maco used for images obtained from the skin sections treated with sodium fluorescein solution, Right: maco used for images obtained from the skin sections treated with Nile red solution.

maco used for images obtained from the skin sections treated with sodium fluorescein solution	maco used for images obtained from the skin sections treated with Nile red solution
// Color Thresholder 1.53k	// Color Thresholder 1.53k
// Autogenerated macro, single images only!	// Autogenerated macro, single images only!
min=newArray(3);	min=newArray(3);
max=newArray(3);	max=newArray(3);
filter=newArray(3);	filter=newArray(3);
a=getTitle();	a=getTitle();
run("RGB Stack");	run("RGB Stack");
run("Convert Stack to Images");	run("Convert Stack to Images");
selectWindow("Red");	selectWindow("Red");
rename("0");	rename("0");
selectWindow("Green");	selectWindow("Green");
rename("1");	rename("1");
selectWindow("Blue");	selectWindow("Blue");
rename("2");	rename("2");
min[0]=0;	min[0]=0;
max[0]=0;	max[0]=0;
filter[0]="stop";	filter[0]="stop";
min[1]=60;	min[1]=0;
max[1]=255;	max[1]=0;
filter[1]="pass";	filter[1]="stop";
min[2]=0;	min[2]=125;
max[2]=0;	max[2]=255;
filter[2]="stop";	filter[2]="pass";
for (i=0;i<3;i++){	for (i=0;i<3;i++){

selectWindow(""+i);	selectWindow(""+i);
setThreshold(min[i], max[i]);	setThreshold(min[i], max[i]);
run("Convert to Mask");	run("Convert to Mask");
if (filter[i]=="stop") run("Invert");	if (filter[i]=="stop") run("Invert");
}	}
imageCalculator("AND create", "0","1");	imageCalculator("AND create", "0","1");
imageCalculator("AND create", "Result of 0","2");	imageCalculator("AND create", "Result of 0","2");
for (i=0;i<3;i++){	for (i=0;i<3;i++){
selectWindow(""+i);	selectWindow(""+i);
close();	close();
}	}
selectWindow("Result of 0");	selectWindow("Result of 0");
close();	close();
selectWindow("Result of Result of 0");	selectWindow("Result of Result of 0");
rename(a);	rename(a);
// Colour Thresholding-------------	// Colour Thresholding-------------
run("Invert");	run("Invert");

Table S2: List of Abbreviations

AF	-	autofluorescence
AF-SC	-	autofluorescence of the stratum corneum
AI	-	active ingredient(s)
AROSA-value	-	intensity (MGV/px) after the removal of the skin`s autofluorescence-value
BP	-	broad pass
LP	-	long pass
MGV/px	-	mean grey value/pixel
MPD	-	mean penetration depth
RSD	-	relative standard deviation
SC	-	stratum corneum
SCT	-	stratum corneum thickness
SD	-	standard deviation
TEWL	-	transepidermal water loss
